# Breeding Ewe Lambs: An Australasian Perspective

**DOI:** 10.3390/ani12223207

**Published:** 2022-11-19

**Authors:** Paul R. Kenyon, Rene A. Corner-Thomas

**Affiliations:** School of Agriculture and Environment, Massey University, Private Bag 11222, Palmerston North 4410, New Zealand

**Keywords:** ewe lamb, reproduction, performance

## Abstract

**Simple Summary:**

There are a number of potential advantages and disadvantages associated with breeding ewe lambs at 7 to 9 months of age. In extensive pastoral systems, such as those in Australia and New Zealand, a relatively low percentage of ewe lambs are bred, which suggests that the decision to breed ewe lambs is based on the perception that the potential advantages outweigh the disadvantages. This review outlines current knowledge of ewe lamb breeding with a focus on more recent Australasian studies, particularly relating to factors that influence breeding success. Differences in reproductive success of ewe lambs and mature ewes are highlighted to help identify where differences occur. Furthermore, management guidelines beginning from the weaning of the young ewe herself, through her first breeding, post-weaning of her first set of lambs, and to her second breeding are outlined. Of particular importance is ensuring that ewe lamb live weight and/or body condition score targets at breeding at 7 to 9 months of age are met and appropriate feeding guidelines are followed throughout pregnancy. Adherence to these guidelines should ensure that reproductive success is high and that the potential disadvantages of breeding ewe lambs are mitigated. The potential long-term effects of breeding ewe lambs are also outlined. The review highlights where knowledge is lacking, with a particular focus on Australasian production systems, and where further research is required.

**Abstract:**

A number of potential advantages have been identified for breeding ewe lambs at 7 to 9 months of age, including increased lifetime productivity and profitability. However, breeding at this young age in extensive pastoral systems, such as in Australasia, can be associated with a number of disadvantages resulting in uptake of this management procedure being relatively low. This review highlights the known differences between ewe lamb and mature ewe reproductive performance, thus differing in their management. The review then summaries the scientific literature of factors that affect ewe lamb reproductive success, with a focus on recent studies conducted under extensive pasture-based conditions in Australasia. In particular, this review outlines the importance of ewe lamb live weight and body condition score on their productivity. The potential long-term consequences of breeding a ewe lamb at a young age in terms of her future success and that of her offspring to weaning are briefly outlined. In addition, the potential impacts of selecting progeny born to ewe lambs as future replacement ewes are discussed. Throughout this review, optimal management guidelines from prior to breeding the ewe lambs until rebreeding at 2 years of age are provided. Lastly, areas requiring future research are identified and discussed.

## 1. Introduction

Breeding ewe lambs (termed hoggets in some countries) to lamb at 12 to 14 months of age is seen by many producers as a means of improving flock productivity and profitability. Breeding ewe lambs reduces the duration that ewes are nonproductive from a reproductive perspective compared with systems where, traditionally, ewes lamb for the first time at 2 years of age. This narrative review uses an unstructured literature search to describe our current knowledge of the productivity of ewe lambs and identify factors that farmers can manipulate to improve performance, particularly from an Australasian production perspective.

Recent bio-economic modeling using an average New Zealand farming scenario reported that, as ewe lamb weaning rate increased, so did overall flock profitability [1]. This finding was based on scenarios with a mature ewe weaning rate of either 132% or 150% whilst maintaining the same annual demand for feed for the flock which required fewer mature ewes to be maintained in the flock [1]. In a further analysis, they reported that the ‘break-even’ economic point for a flock with a mature ewe weaning rate of 135% occurred when the ewe lamb weaning rate was at least 26% [2]. This suggests that breeding ewe lambs can be profitable even when only a relatively low proportion of ewe lambs are successfully bred. The analysis also showed, however, that, before focusing on ewe lamb breeding, it may be more profitable to increase mature ewe reproductive performance if it is low [2]. Overall, the most profitable scenarios occurred when there was high reproductive performance of both mature ewes and ewe lambs.

Modeling undertaken under Australian conditions [3,4] also reported that there was potential for ewe lamb breeding to lift overall farm profitability. Young and others [4] also suggested that there would be considerable return on research investment if research was focused on lifting ewe lamb reproductive performance. More recently, Tocker and others [5] reported that successfully breeding ewe lambs increased profit and reduced business risk. Furthermore, they found that the type of herbage offered to ewe lambs influenced the potential financial success of ewe lamb breeding by lifting fertility rates (ewe pregnant per 100 ewes bred).

In Australasia, sheep are generally managed in extensive pasture-based systems where sheep remain outdoors all year round [6,7,8]. Within these systems, there is a high degree of seasonality due to seasonal breeding activity of the breed types and variation in pasture growth due to temperature and rainfall patterns. In New Zealand, the diet of sheep is made up of approximately 95% pasture with a small amount of supplementation provided in the form of grazed crops and conserved pasture. In Australia, the rate of supplementation has been estimated to be 45% of total domestic feed usage due to greater periods of pasture senescence and greater access to locally grown grain [9].

Potential further advantages from breeding ewe lambs successfully can include improved utilization of spring-grown herbage through greater overall flock demand, increased number of lambs weaned per year, an early ewe replacement selection tool, decreased generation interval if replacements are selected from those born to ewe lambs, increased selection pressure through more potential replacement ewes, and improved greenhouse gas efficacy per kg of product [10,11]. Some potential limitations and disadvantages, however, have been identified. These can include variable reproductive performance, the potential for increased flock feed demand, heavier live weights required at 7 to 9 months of age, potential negative lifetime effects if ewe liveweight is negatively affected, lighter lambs at birth and weaning, poorer progeny survival, increased total farm costs and workload, reduced ewe lamb wool production, and the potential for greater ewe lamb mortality [10]. These potential limitations and disadvantages likely drive the low percentage of ewe lambs bred (~30%) in both New Zealand and Australia [12,13]. This review, therefore, outlines our current knowledge of factors that influence ewe lamb productivity and that drive success, with a focus on more recent Australasian pastoral-based research. Furthermore, the potential long-term implications of ewe lamb breeding and current knowledge gaps are discussed. This review, where appropriate, also refers to previous reviews of ewe lamb breeding [10,11,14,15].

## 2. Comparison of Reproductive Traits of Ewe Lambs and Mature Ewes

It is accepted that the reproductive performance of ewe lambs is consistently lower than that of both 2 year old and mature ewes [10]. A recent survey of farmers in Australia found that the reported reproductive rate of ewe lambs and mature ewes differed by 50% [16]. The lower number of lambs weaned per ewe lamb presented for breeding is due to a number of factors (Table 1). These factors include later onset of breeding activity within breeding season, less intense estrus behavior, reduced ovulation rate, poor breeding behavior, greater embryonic and fetal mortality, lower lamb birth weights, and reduced lamb survival. Combined, these factors also result in highly variable reproductive success. In a large-scale study of commercial flocks in New Zealand, fertility rates (ewes pregnant per 100 ewes bred) ranged between 77 and 92, and fecundity rates (number of fetuses per 100 ewes bred) ranged between 108 and 142 [17]. Furthermore, farmer self-reported fecundity rates were even more variable ranging from 10% to 130% [18]. Few studies have directly compared ewe lamb and mature ewe reproductive success when bred and managed together until weaning of their lambs [19,20]. The majority of studies comparing reproductive performance of mature ewes and ewe lambs are based on either industry data or studies in which mature ewes and ewe lambs were not bred and managed together, which limits the accuracy of the comparison (Table 1).

## 3. Factors Affecting Ewe Lamb Performance Prior to and during Breeding

### 3.1. Genetic Factors

Genetics can influence ewe lamb reproductive performance. Traits known to be under genetic influence that then impact ewe lamb performance include age and body weight at puberty, and the number of estrous cycles within the season [67]. In addition, the number of ewe lamb estruses and fertility as a 2 year old are also genetically related [68,69]. Heritability data for many ewe lamb reproductive traits are sparse, but knowledge is increasing. Overall, the available data suggest that the heritability of traits such as age and live weight at puberty [70,71], number of cycles in the first year [72], fertility, fecundity, and number of lambs born and weaned by ewe lambs are low to moderate at best [73,74], indicating that genetic gains can be made but will be slow. Newton [75] indicated that the fertility of ewe lambs was correlated with their later reproductive performance; however, they were genetically different traits. Rosales Nieto and others [76,77] reported that ewe lambs with higher breeding values for growth post weaning or fat and eye muscle area, or whose sires had greater breeding values for these traits were younger at puberty, more likely to achieve puberty by 8 to 10 months, and more fertile, had lower rates of pregnancy loss, and achieved a higher overall reproductive performance. Similarly, Thompson and others [78] reported that ewe lambs from sires with higher breeding values for fat achieved higher fertility and reproductive rates. Rosales Nieto and others [79] reported no effect of higher breeding values for growth post weaning or fat and eye muscle area on ewe lamb milk production but observed faster growth to weaning among progeny. Similarly, Paganoni and others (2014) reported that pregnancy failure was lower in ewe lambs whose sire had higher breeding values for weight and fat post weaning. As more heritability data, genetic correlations, and impacts of sire type are collated, it is expected that farmers will use this information when selecting appropriate sires. Newton [80] reported that consideration of genomic information when selecting replacement ewes lambs at 7 months of age decreased the risk for breeding programs.

Variation exists among breeds and genotypes with regard to timing and live weight at puberty, proportion of ewe lambs displaying estrus (i.e., puberty achievement) within season, length of reproductive season, pregnancy and reproductive rates, and lambing percentage [10,16,81]. Therefore, farmers have scope to select breeds or genotypes most suitable to be bred as ewe lambs.

#### Potential for Fetal Programming Effects

In sheep, there is some evidence to indicate that early life perturbation can have long-term impacts, which is sometimes referred to as fetal programming [82]. In cattle, the number of follicles a heifer is born with is affected by both environmental and genetic factors [83]. Furthermore, poor dam nutrition and health issues such as mastitis can negatively affect the progeny’s final follicle number and reproductive function as an adult [84]. In sheep, there appears to be few studies that examined the impacts of the management of the dam during both pregnancy and lactation on reproductive performance of the resulting progeny as a ewe lamb. Furthermore, the impact of nutrition around the time of weaning and post weaning on ewe lamb reproductive and lactational performance is limited.

### 3.2. Environmental Factors

#### Timing of Puberty and Breeding

In seasonal breeds, there is a limited period in which ewe lambs can be successfully naturally bred. This limitation is due to the necessity for ewe lambs to achieve puberty during the breeding season [33], thus placing an additional constraint on ewe lamb breeding success. The majority of breeds farmed in Australasia are seasonal and cannot naturally be bred year-round.

The breeding season tends to be shorter in ewe lambs compared to mature ewes, as the onset of puberty generally occurs much later in the breeding season than the natural onset of mature ewe breeding activity [21,22,25,85]. This delayed onset of reproductive activity in ewe lambs can result in many ewe lambs only achieving one or two estrus events before seasonal anestrus occurs [14]. Foote and others [28] reported that ewe lamb ovulation rate did not generally increase over the first to third estrous cycles, although Hare and Bryant [26] found that fertility rates increased in each subsequent cycle from the puberal estrus.

Time within the breeding season also influences ewe lamb breeding success [26,86]. Reproductive performance in ewe lambs is not only influenced by day length, but also requires that they be physiologically mature enough to achieve puberty [87]. This is driven by hormonal factors and influenced by muscle and fat accumulation [88]. Combined, these findings indicate the importance of ensuring ewe lambs reach puberty as early as possible, within the natural breeding season, if the aim is to maximize the numbers successfully bred. Edwards and others [89] reported that two-tooth reproductive performance was also greater in those that achieve puberty as a ewe lamb, further reinforcing the importance of achieving puberty in the first year of life. Furthermore, Wall and others [90] reported increased ewe lifetime economic performance among ewes first bred at 18 months of age when they attained puberty as a ewe lamb (in their first year of life) compared to those that had not. Thus, knowledge of factors ensuring early onset of puberty and methods to manipulate the onset of puberty are discussed in later sections.

**Figure 1:**
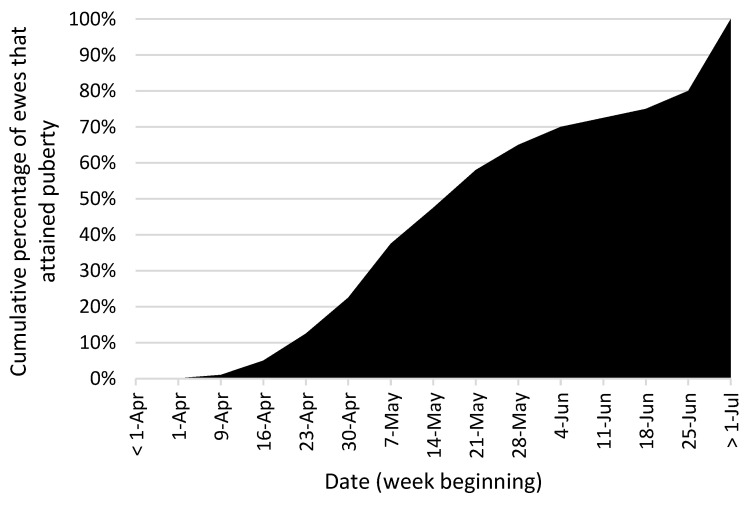
The cumulative proportion of ewes that attained puberty each week of the breeding period in the South Island of New Zealand (adapted from [11]). Note: In New Zealand, mature breeding begins in March/April, and the shortest day occurs on 21 June.

Edwards and Juengel [11] reported that, in New Zealand, while some ewe lambs achieved puberty in early April (Figure 1), it was not until late May that 50% achieved puberty. Under New Zealand conditions, ewe lamb breeding traditionally begins in early May, approximately 1 month after mature ewes [11]. Given the longer-term aim on most farms to re-breed ewe lambs at approximately 18 months of age, at the same time as the mature ewe flock, early achievement of pregnancy maximizes the time the young dam has to recover prior to rebreeding.

Age influences when a ewe achieves puberty; however, it is often confounded by live weight [33,91]. Recently, it was reported that the relationship between age at breeding and reproductive rate between 6 and 9 months of age was linear [92]. Therefore, in a once-a-year lambing system, e.g., in spring, an earlier-born lamb is more likely to achieve puberty in the subsequent autumn. There is a general lack of data that separate the potential impacts of age and live weight. Rosales Nieto and others [15,76] reported that faster growth pre and post weaning can result in the ewe lamb reaching puberty at a younger age, although at a slightly heavier live weight. It has, therefore, been suggested that farmers should select first-born lambs within the lambing season as replacements for ewe lamb breeding [76]. However, under many extensive commercial farming systems, farmers do not collect data on individual birthing dates, making this approach difficult. Farmers, however, could utilize mating pattern data or ultrasound scanning to ensure early lambing ewes are lambed separately and their progeny preferentially selected as replacements. The optimal timing of breeding of a ewe lamb is also affected by both location and genotype [33,93].

### 3.3. Management Factors

#### 3.3.1. Exogenous Hormones and the Ram Effect

Puberty can be advanced though the use of either exogenous hormones or the male (ram) effect, but both have limitations [94,95]. The use of exogenous hormonal regimens in lightweight ewe lambs that are not physiologically mature places them at greater risk of failure. Therefore, these are not advocated as a tool to either advance puberty or increase pregnancy rates in ewe lambs. In ewe lambs that have reached target live weights, there may be a place for the use of exogenous hormones; however, there appears to be a lack of research examining their effects and their cost effectiveness.

The ram effect, using vasectomized rams, can be used to advance the breeding date, proportion of ewe lambs bred in the first 17 days of breeding, and overall pregnancy rates [96,97]. A ratio of vasectomized rams to ewe lambs of 1:70–100 has been suggested [98]. Vasectomized rams should be used in the 17 day period directly prior to planned start of breeding [99]. Potential alternatives to vasectomized rams include short-scrotum ram lambs or a short exposure of a few days to mature rams [99,100]. The ram effect should not be used as a tool to induce puberty in lightweight ewe lambs, as this can increase the risk of reproductive failure in later stages of pregnancy and lactation or in future years. An alternative use of vasectomized rams is to identify ewe lambs that achieve puberty, but are not subsequently bred, as a screening tool to select more fertile/fecund replacements in traditional systems where ewes are bred for the first time at an older age [89].

#### 3.3.2. Effect of Liveweight, Liveweight Change, and Body Condition Score (BCS) prior to and during Breeding

**Figure 2:**
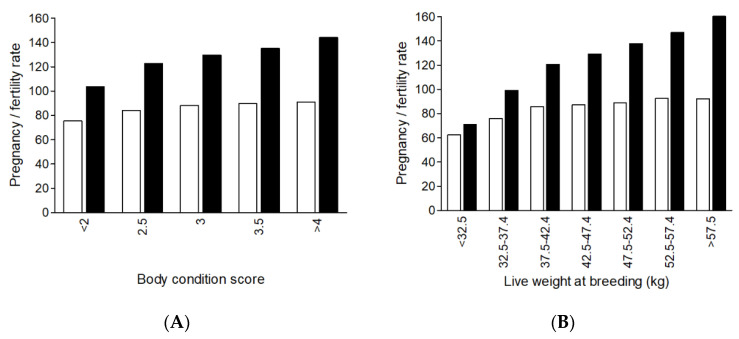
The effect of ewe lamb body condition score (**A**) and breeding liveweight category (**B**) on ewe lamb fertility rate (ewes pregnant per 100 ewes presented for breeding; white bars) and reproductive rate (number of fetuses per 100 ewes presented for breeding; black bars). Adapted from [17].

Within a given genotype, live weight is likely the biggest driver of the reproductive success of ewe lambs. It is difficult to separate the impacts of liveweight and liveweight change just prior to and during the breeding period on ewe lamb reproductive performance. In fact, few studies of ewe lambs have even attempted to do this. It is well established that there is a positive relationship between ewe lamb liveweight and most reproductive traits [10,11,40,42,88,92,101,102]. Thus, any factor that influences the growth of a ewe lamb from her conception until the end of her first breeding period will impact reproductive success. The impact of live weight on reproductive success is not linear, with a plateauing relationship (Figure 2) [17,42,78,92]. Recently, in maternal composites, Thompson and others [92] reported curvilinear relationships for both reproductive rate and weaning rate responses with breeding liveweight, with a plateau at approximately 45 kg. This is similar to the fertility data reported by Corner-Thomas and others [101] in Romney ewe lambs. On the other hand, in the subset of Merino ewe lambs included in an Australian study, their suggested plateau was around 50 kg [76].

Body condition score (BCS) is known to generally have a diminishing returns relationship with most reproductive traits among mature ewes [103], although less is known for ewe lambs. BCS in ewe lambs may be limited by the fact that a young animal is more likely to deposit lean (muscle) rather than adipose (fat) tissue [104]. Among Romney ewe lambs reproductive performance increased with BCS, albeit in a diminishing returns relationship with a plateauing effect at a BCS range of 3.0 to 3.5 (Figure 2; [17]). It has been suggested that a ewe lamb that has greater levels of fat is more physiologically mature and more likely to achieve puberty and be bred successfully [15,105]. Corner-Thomas and others [17] suggested that, for ewe lambs, a minimum target BCS should be 3.0 at breeding.

It has been well established in ruminants that puberty occurs when individuals reach 40–65% of their mature liveweight [104]; therefore, within breed and genotypes, there are differing minimum liveweight targets. In New Zealand, for example, current industry best practice guidelines for Romney-type ewe lambs is a minimum liveweight of 42 kg at breeding [106]. On the other hand, Corner-Thomas and others [17] suggest that the minimum weight in Romneys should be 47.5 kg if the aim is to maximize reproductive performance. In Australian flocks, Rosales Nieto and others [15] suggested that Merinos should have a minimum liveweight of 40 kg. Using minimum guidelines, farmers should monitor the liveweight of their ewe lambs from weaning at 3–4 months of age to ensure they achieve the minimum breeding weight. Early identification of ewe lambs with liveweights below target allows for the implementation of targeted feeding to increase liveweight gains, thereby ensuring targets are met.

**Figure 3:**
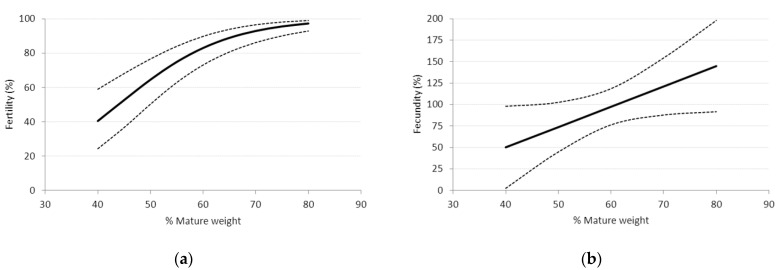
The relationship between percentage of mature weight and (**a**) fertility and (**b**) fecundity of 8 month old Romney crossbred ewe lambs (logit predictions and 95% confidence intervals are shown; adapted from [107]).

Kenyon and others [107] examined the impact of breeding weight of Romney crossbred ewe lambs as a percentage of mature weight (mean 4 year old breeding weight = 63 kg). They found that, as the percentage of mature weight increased, so did fertility in a curvilinear manner, which plateaued at approximately 70% of mature weight (Figure 3). If this relationship was to hold in all breeds and genotypes, it would allow farmers to target a minimum 70% of mature weight when selecting ewe lambs for breeding.

The growth and development of the ovarian follicle and its maturation to ovulation take around 6 months [108]. Ovum quality is affected by the environment that the young ewe lamb is subjected to in early life and potentially the in utero environment [43,49]. Therefore, aiming only for a rapid gain in liveweight just prior to breeding to ensure that the target liveweight is met is not likely to be the optimal strategy.

There is sparse research on the potential for ewe lambs to respond to the ‘flushing effect’ prior to breeding, which is well established in mature ewes [109,110]. Corner-Thomas and others [111] showed that a 1 month period of improved nutrition by offering pure lucerne sward rather than ryegrass white clover pastures had the potential to improve twinning rates, but this was likely dependent on feed quality.

Nutrition during the breeding period is positively related to both ewe lamb fertility and reproductive rates. Rosales Nieto and others [105] showed that gaining liveweight, through improved feeding, compared to maintaining liveweight during the breeding period increased both indicators of reproductive performance. In support of this, Thompson and others [78] showed that liveweight gain during breeding also increased these indicators. They suggested, however, that the positive impacts of liveweight gain on reproductive performance were less apparent at heavier mating live weights. They also noted that through greater liveweight gains, the overall reproductive performance of lighter ewe lambs at breeding could be lifted to be similar to those that were heavier at the start of breeding. Mulvaney and others [112] also reported the potential for a positive influence of liveweight gain during breeding. Combined, these data indicate the importance of feeding ewe lambs well during the breeding period to ensure they are gaining liveweight.

#### 3.3.3. Shearing before and during the Breeding Period

The impacts of shearing ewe lambs before breeding on reproductive performance have been somewhat inconsistent [10]. However, combined, the results suggest that shearing should be avoided within 4 weeks of breeding and during the breeding period. Shearing within this window can result in delayed onset of puberty due to stress [113], as well as depressed ovulation rate and consequent lower pregnancy and multiple bearing rates [114]. However, under warm and humid environmental conditions during the summer and autumn period, it has been suggested shearing can alleviate heat stress and, thus, stimulate appetite and liveweight gain [10]. Therefore, in this situation, shearing should occur well before the breeding of ewe lambs and, in some environments, could be utilized as a management tool to help ensure that appropriate breeding live weights are achieved.

#### 3.3.4. Breeding Management

As outlined in Table 1, ewe lambs are in estrus for a relatively short period and are less likely to seek the ram and stand for him compared to mature ewes, all of which individually, let alone combined, reduce pregnancy rates [22,33]. During the breeding period, ewe lambs and mature ewes should not be bred together as the mature ewes have more intensive breeding behavior and will dominate the ram’s attention [65]. More ‘ram’ power is required than for mature ewes. It has been suggested under pastoral extensive conditions that mature ram-to-ewe lamb ratios should be in the range of 1:50 to 1:75 [98], compared to 1:100 to 1:150 in mature ewes. The absolute ratio is likely to be dependent on the size of the paddocks and their topography.

Ram lambs can be utilized to reduce the generation interval, if the resulting progeny are kept as replacements, or as an option to reduce ram costs. However, when utilizing ram lambs, lower ram-to-ewe ratios than recommended for mature rams are required [115]; otherwise, pregnancy rates may be disappointing. Alternatively, 18 month old rams can be just as effective as mature rams [115], and the reuse of mature rams directly after being bred with mature ewes is another option to reduce ram costs [116].

As indicated earlier, heavier liveweights at breeding are associated with positive impacts on ewe lamb breeding performance [10,92]. Therefore, improved levels of nutrition during the breeding period resulting in greater liveweights should have a positive effect on conception and pregnancy rates [10,11]. Interestingly, in synchronized ewe lambs, there is some evidence that very high liveweight gains (170 g/day plus) can be associated with higher returns to service rates in the first cycle of breeding, although, overall pregnancy rates tend not to differ [112,117]. This may suggest that, while farmers should ensure ewe lambs are gaining liveweight prior to and during the breeding period, they should avoid very high liveweight gains.

## 4. Factors Affecting Ewe Lamb Performance during Pregnancy

### 4.1. Nutritional Management

Pregnancy losses are greater in ewe lambs than in mature ewes (Table 1), although the causes of many of these losses are poorly understood. There is some evidence, however, to suggest that nutrition of the young dam plays a role [10]. The majority of pregnancy nutrition studies have occurred either under New Zealand’s pastoral conditions or in the UK under indoor conditions with concentrate feeds. Indoor studies have often been models for human studies and have generally involved very young ewe lambs (6 to 7 months), concentrate supplements, embryo transfer, and liveweight gains in excess of 200 g/day [118,119,120,121,122], which exceed those generally observed under pastoral conditions. Therefore, caution is required when extrapolating these results to pasture-only grazing conditions in production systems such as in Australia or New Zealand. These relatively high liveweight gains have been found to negatively affect pregnancy maintenance (i.e., cause fetal loss), fetal growth, birth weight, ewe and fetal metabolism, colostrum yield, and lamb survival. In other indoor studies with a production focus, lower liveweight gains have not resulted in the same effects (see review by Kenyon and others [10]).

The majority of pastoral studies reported no association of liveweight, liveweight gain, or body condition during pregnancy with ewe lamb pregnancy status or lamb birth weight [123,124,125,126]. On the other hand, under pasture-only grazing conditions, pregnancy feeding resulting in liveweight gains of approximately 180 g/day or greater for long periods of pregnancy have tended to result in higher rates of pregnancy loss rates and/or reduced lamb survival [127]. Very few pastoral studies under New Zealand conditions have achieved liveweights near or above 200 g/day [112], and none have consistently reported gains throughout pregnancy in excess of 250 g/day [10]. Liveweight gains greater than 250 g/day in ewe lambs are possible under pastoral grazing conditions; therefore, it would be of benefit for future studies to consider the potential impacts of liveweight gains above 250 g/day during breeding and pregnancy. In farming systems such as in Australia, ewe lambs can be potentially offered a mix of herbage and grain-based concentrates resulting in high liveweight gains.

Concentrate feeding studies in the UK that resulted in the ewe lamb losing conceptus-free live weight in pregnancy reported reduced fetal growth, lamb birth weight, and colostrum production [50,128]. Under New Zealand conditions, low ewe lamb liveweights prior to breeding and/or low liveweight gains in pregnancy are risk factors for pregnancy and fetal loss [127,129] (also see Section 4.2).

Mulvaney and others [127], and Ridler and others [129] reported that lower weights from breeding through to and/or including early pregnancy or lower liveweight gains in early pregnancy were associated with higher rates of pregnancy loss. Furthermore, there are data indicating that ewe lambs who lose conceptus-free liveweight in pregnancy are more likely to have subsequent fetal loss [130] or fail to rear a lamb [131]. However, it is not known if this is a cause-and-effect relationship, or if the loss in conceptus-free liveweight occurs as a result of pregnancy loss.

Current New Zealand pastoral-based recommendations suggest that farmers should aim for ewe lamb liveweight gains throughout pregnancy in the range of 120 to 150 g/day [106]. These recommendations are based on meeting the expected conceptus mass weight at term, plus allowing for the young dam herself to grow, especially in the first two-thirds of pregnancy [10]. By continuing to grow herself in pregnancy, the young dam will also be better prepared for parturition and lactation, which allows for an easier transition to rebreeding. Kenyon and others [123] suggested total liveweight gains of approximately 200 g/day are less efficient in terms of kg feed consumed per kg of ewe lamb liveweight, indicating that, from a productive efficiency perspective, there may be a fine line between under- and overfeeding.

Ewe lamb live weight at all stages of pregnancy can have a small positive impact on the weight of her lamb(s) at weaning [127,132]. Modeling has shown, however, that liveweight of the ewe lamb at breeding compared to the various stages of pregnancy has the greatest impact on both her own liveweight and that of her offspring at weaning [133]. For example, for every additional kg of ewe lamb live weight prior to breeding, there was an additional 326 g of lamb weaned for single-rearing ewes compared with 106 g for the same liveweight difference at 150 days of gestation. This further supports the importance of achieving suitable breeding weights. Similarly, Thompson and others [78] reported that liveweight gain during breeding had no impact on lamb birth weight but a small positive effect on weaning weight.

The sparse data available suggests that the BCS of the ewe lamb in pregnancy has no impact on the weight of offspring at weaning. Studies of BCS during pregnancy among mature ewes have been inconsistent, with reports of no effect or a positive relationship with lamb weaning weight. The low number of studies available and the potential interaction with feeding levels limit our ability to clearly determine the impact of BCS on the weaning weights of ewe lamb progeny. There is some suggestion, however, that high BCS in pregnancy may have negative effects on lamb birth weight [101].

### 4.2. Pregnancy and Fetal Loss

The level and cause of fetal and pregnancy losses have been examined on numerous occasions in New Zealand [112,123,124,125,129,130,134,135,136,137]. In addition, a recent large-scale study in Australia on commercial flocks reported significant pregnancy loss rates but results were highly variable across farms, ranging between 0% and 48% [126]. The potential impacts of nutrition, liveweight, and BCS were covered in the previous section. This section, therefore, focused on other potential causes of fetal and pregnancy loss.

Abortive endemic diseases can play a role in pregnancy loss (abortion) in ewe lambs [134,135,138]. Young ewes are more susceptible, as they are less likely to have developed immunity compared to an older ewe [139]. Identifying risk factors contributing to in utero losses is difficult, as multiple factors may be involved. In one New Zealand study, low liveweights at breeding and early pregnancy were associated with increased mid- to late-pregnancy abortion [129]; however, in another study, the same group reported no effect of liveweight [130]. Clune and others [126] suggested that, according to a series of Australian studies by their group, diseases such as *Chlamydia pecorum*, *Campylobacter fetus* fetus, *Toxoplasma gondii*, *Neospora caninum*, and *Coxiella burnetiid* had the potential to cause abortion on farms but were not large contributors to industry-wide losses. Similarly, in New Zealand, *Neospora caninum* [134,135] and *Leptospira* serovar Pomona appear not to be major causes of loss [129]. Kenyon and others [18], however, reported a positive lambing percentage response in ewe lambs from vaccination against toxoplasmosis and campylobacteriosis.

Further disease investigation and use of tools such as serial pregnancy scanning are warranted to gain a further understanding of causes and the level of pregnancy loss in ewe lambs. Differentiating losses in pregnancy from perinatal loss, and determining the causes of these losses will help inform strategies to improve overall reproductive performance in ewe lambs [126].

### 4.3. Mid-Pregnancy Shearing

Mid-pregnancy shearing of mature ewes between approximately days 50 and 110 of pregnancy, under pastoral conditions, is an established management tool to positively increase both lamb birth weight and survival, especially in multiples [140]. However, far less research has been undertaken with ewe lambs. The few studies that have been conducted suggest that mid-pregnancy shearing can increase singleton lamb birthweight, but not twins, and it did not influence survival [141]. Given the potential risk of increased dystocia, through heavier lamb birthweights in ewe lambs, care is required when utilizing this management option.

## 5. Factors Affecting Performance during Lambing and Lactation

### 5.1. Lamb Survival

Industry data often suggest that perinatal lamb losses are far greater in ewe lambs than mature ewes [39,110], although few studies have directly compared losses when the two age classes have been lambed together due to differences in the timing of breeding. Studies which did not directly compare the two ewe age classes suggested higher rates of lamb loss (12% to 60%) among ewe lambs compared to mature ewes [112,127,129,130]. Although the sparse direct comparison data available indicate only a slightly higher rate of loss, this was not always the case [142].

Ridler and others [143] reported that the greatest cause of death for lambs born to ewe lambs was stillbirth (~40%), followed by starvation exposure (26%) and dystocia (17%). They identified that low lamb birth weight, multiple litters, and increasing ewe lamb weight gain from breeding to late pregnancy were risk factors for both lamb mortality and stillbirth. This finding was unexpected as Griffiths and others [131] reported that low non-conceptus free live weight gains in pregnancy were associated with an increased risk of a ewe lamb failing to successfully rear a lamb. The difference in findings may be due to the liveweight gain profile and the way liveweight was determined between studies. Hinch and Brien [144] indicated that, in mature ewes, adequate feeding levels during pregnancy reduced the risk of starvation exposure by helping ensure that lambs were of suitable birth size and that the young dam was producing suitable levels of colostrum and milk. Thompson and others [78] reported that changes in ewe lamb liveweight over the breeding period and in the very early stages of pregnancy had no impact on lamb survival.

Dystocia (or birthing difficulty) has consistently been reported to be one of the major causes of lamb mortality [145,146]. Dystocia is predominantly driven by the pelvic opening not being large enough to expel the fetus; this is especially an issue if the ewe lamb is not well grown [145,147]. Farmers can mistakenly think they can reduce feed intake in later stages of pregnancy to mitigate against this by controlling lamb birth size. However, this will not significantly influence lamb birth size/weight, as these are predominately driven by the genetic makeup of the fetus and the genetic ability of the ewe lamb to partition nutrients to the placental–fetal unit [147]. To limit dystocia, farmers need to ensure that ewe lambs are well grown through appropriate feeding levels prior to and during pregnancy to ensure that the young dam continues to grow and is structurally large by lambing [106]. Furthermore, appropriate sire choices are needed, although there appears to be sparse data on the best genotype and/or breeds to utilize as sires and impacts of genetics on birth size and dystocia. From a lamb survival perspective, the optimal lamb birth weight range of lambs born to mature ewes across birth ranks has been well characterized [144,148]. However, these data appear to be sparse with regard to lambs born to ewe lambs.

Ridler and others [143] noted that, over the lambing period, death of the ewe lamb accounted for 11% of lamb deaths. Their results also indicated the importance of monitoring ewe lambs over the lambing period to assist not only newborn lambs to ensure adequate survival rates, but also the young dam. The decision to monitor lambing, however, should be carefully considered as primiparous ewes are more reactive to disturbance and generally show poorer maternal behavior than multiparous ewes [149].

### 5.2. Nutrition

Lactation is a period in which the young dam needs to continue to grow to meet later liveweight targets and maximize milk production. There are potentially long-term impacts of nutrition during lactation on the liveweight and condition of the young dam going through to her next breeding. It is, therefore, vital that intakes should not be restricted during this period. Very few studies, however, have examined the impact of feeding levels in lactation on ewe lamb performance and that of her offspring. In New Zealand, alternative forages such as herb–clover mixes and pure lucerne swards have been shown to increase the liveweight of the both the young dam and her lambs at weaning compared to ryegrass-white clover pastures [150,151].

### 5.3. Lamb Age at Weaning

Due to the potential conflict between the energy cost to rear lambs and the need for energy to allow continued growth of the ewe lamb, early weaning is an option. This is of particular importance in production systems where ewe lambs are bred after the mature ewe flock, but their second breeding period, post 1 year of age, is at the same time. Early weaning in this scenario would reduce the nutrition demand on the young dam and give her more time to reach a suitable breeding weight. Mulvaney and others [152] reported that weaning ewe lambs from their lambs at 10 compared to 14 weeks resulted in greater liveweight gains in this period for the young dam, without detrimentally affecting the weight of their lambs. In that study, the number of lambs was relatively low (<100), and lamb survival was not reported.

### 5.4. Post-Weaning Management

There is sparse information on the impacts of management, including nutrition, of the ewe lamb between weaning and her next breeding at 18 months of age. This period is a window of opportunity to potentially remediate poor liveweight and/or body condition due to pregnancy and lactation. Given the established relationships between both live weight and body condition with reproductive performance in older ewes [9], it would be expected that any management tools ensuring that the young ewe continues to gain liveweight and BCS post weaning of her lambs will have positive effects.

## 6. Lifetime Impacts of Ewe Lamb Breeding

In the literature, reports of the impacts of breeding ewe lambs on 2 year old and lifetime performance are inconsistent. Most studies have been undertaken under New Zealand and UK conditions. Many studies reported lighter liveweights at 18 months after ewe lamb breeding; however, in most studies, the impact was relatively small [10]. Furthermore, any impact on two-tooth liveweight generally did not persist past the weaning of the second set of lambs [10]; however, in some studies, it took until after the third set of lambs [153]. Breeding a ewe lamb can negatively affect her fleece weight until approximately 18 months of age, but no longer-term impacts have been reported [52,154,155].

Flay and others [156], using data from a large-scale lifetime study on commercial farms in New Zealand, reported that there was no effect of pregnancy or rearing a lamb on the risk of a ewe being subsequently culled or being identified as dead or missing [156]. They did, however, report that increased BCS at breeding as a ewe lamb or at 18 months was associated with reduced ewe wastage in that reproductive year. Thomson and others [153] reported ewe lamb losses during lambing resulted in fewer ewes remaining in the flock in future years compared to ewes that lambed for the first time at 2 years of age. Combined, these data indicate the importance of appropriate feeding and management of ewe lambs both prior to breeding and in pregnancy and lactation to ensure that rising 2 year breeding weights and longevity are not negatively affected.

Thomson and others [153] reported that ewes that first lambed at 1 year of age, compared to 2 years weaned more lambs in their lifetime, but individual lamb weaning weight did not differ, resulting in greater total weight of lamb weaned. This matches the previous finding of increased fetuses per lifetime of ewes that lambed at 1 year of age [157]. In both studies, the reproductive performance achieved by breeding at 7 to 9 months of age was a major driver of the overall greater lifetime reproductive performance. This suggests that, if breeding at a young age is to be successfully utilized by farmers, management practices must be in place to ensure high reproductive performance in the young ewe. Therefore, the entire potential lifetime success of ewe lamb breeding, in terms of performance, is dependent on nutritional management in the first 18 months of life. The results of Kenyon and others [157] suggest that, when appropriate two-tooth liveweight and body condition score targets were met, lifetime reproductive performance of ewes bred as a ewe lamb were greater than those that were not bred.

While liveweight at breeding as a ewe lamb has been shown to impact performance in the weaning of her first lamb, few studies have examined potential long-term effects of liveweight itself at ewe lamb breeding. Haslin and others [158] reported that heavier ewe lamb breeding weights did not influence 2 and 3 year old reproductive performance. However, the analysis of pooled data by Haslin and others [159] showed that heavier ewes at breeding at 8 months of age weaned a greater number of lambs over a 3 year period. However, due the ewe lamb herself being heavier, lamb production efficiency (kg lamb weaned per kg of ewe lamb presented for breeding) did not differ [102,159]. This may suggest some lifetime performance benefits in terms of total weight of lambs weaned resulting from heavier liveweights at ewe lamb breeding.

## 7. Potential Impacts of Selection of Progeny Born to Ewe Lambs as Replacement Ewes

The vast majority of studies comparing the performance of progeny born to ewe lambs or mature ewes finish at weaning (see Table 1). There is currently sparse information on the long-term consequences of selecting progeny born to ewe lambs as replacement ewes. Craig [160] reported that female progeny of ewe lambs had lower reproductive performance when bred, compared to those born to mature ewes. However, many studies did not compare reproductive performance of progeny born to mature ewes or ewe lambs until breeding (at least 18 months of age). Loureiro and others [161,162] reported that the progeny born to ewe lambs were lighter until 1 year of age, but their reproductive performance at 18 months did not differ from those born to mature ewes. In addition, their lactational performance did not differ. There was also no difference in any productive parameters at 3 years of age. In a lifetime study comparing ewes born as either a single or twin to ewe lambs or mature ewes, Pettigrew [163] reported that those born as a twin to ewe lambs were lighter throughout their lifetime than the other three groups. However, among the four groups, there was no difference in total lifetime litter weaning weight.

In a more recent study, in a different cohort, Pettigrew and others [164] investigated the reproductive performance of single and twin ewes born to ewe lambs and twins born to mature ewes at 2 years of age. In that study, ewe lambs that reached the target pre-breeding weight were bred for the first time at approximately 8 months of age. They reported that single and twin ewes born to ewe lambs remained lighter than twins born to mature ewes, with twins born to ewe lambs being the lightest. There was no difference in reproductive performance at 8 months of age of those presented for breeding, although, as fewer twins born to ewe lambs were presented for breeding due to their lower liveweights, their overall reproductive performance was lower. At 18 months of age, there was, however, no difference in reproductive performance. In both years, the weaning weights of lambs did not differ. Combined, these data suggest that, if ewe lambs are heavy enough when bred at 8 months of age, their reproductive performance does not differ from those born to mature ewes. However, the issue is in ensuring that these liveweight targets are met.

## 8. Knowledge Gaps

This review outlined our current knowledge and identified several areas that would benefit from further research. It is possible that, for specific breeds, genotypes, and environments, there would be benefit in confirming the published findings.

Areas identified that warrant further research are as follows:

- Potential long-term impacts in utero experience on ewe lamb reproductive performance (fetal programming),
- Optimal liveweight gain profile for ewe lambs from weaning at 3 months of age until the start of their first breeding period,
- Disease investigation using serial pregnancy scanning to differentiate losses in pregnancy from perinatal loss and causes of these losses,
- Optimal lamb birth weight range for survival of ewe lamb progeny,
- Effect of BCS during pregnancy on ewe lamb progeny weaning weight,
- Optimal nutrition during lactation and the interaction with nutritional levels in pregnancy,
- Management during lactation and post weaning on rebreeding at 18 months of age,
- Lifetime studies or analysis of existing data on ewe lamb lifetime performance,
- Economic analysis of the most cost-effective nutritional regimens in both pregnancy and lactation. This will then allow the identification individual management factors that will help farmers focus their efforts on those that have greater impacts on profitability.

## 9. Conclusions

This review highlighted that breeding ewe lambs can improve lifetime ewe performance and be profitable. Within genotype, drivers of performance are known; in particular, ewe lamb liveweight targets at breeding and nutrition during pregnancy are key factors that can significantly influence not only ewe and lamb performance to weaning but also ewe lifetime performance. Although there has been considerable recent research under Australasian conditions, there are still a number of areas requiring further research. These include the selection of ewe lamb progeny as replacements and the impact of management during lactation and post weaning on rebreeding at 18 months of age. If these issues can be addressed, the uptake of ewe lamb breeding should be increased under extensive pastoral grazing conditions such as those in Australasia.

## Figures and Tables

**Table 1:** Comparison of ewe lamb and mature ewe reproductive traits.

Trait	General Comments in Comparison with Mature Ewes	References
Onset of breeding activity within breeding season	Later due the need to achieve puberty, which is driven by achieving appropriate live weight prior to cyclic activity	[14,21,22,23,24]
Length of breeding season	Shorter due to delayed onset of start of breeding activity within the breeding season window	[14,23,25,26,27,28,29]
Regularity of estrus length	More likely to be irregular	[26]
Length of estrus period and chance of silent estrus	Shorter estrus period. More likely to have estrus without ovulation or ovulation without estrus.	[30,31,32]
Mating behavior	Less likely to seek and stand appropriately for the ram. It has also been suggested that rams prefer mature ewes.	[22,33,34]
Suggested ram-to-ewe ratio	A lower ram-to-ewe ratio required, at least half	[18,22,35,36,37]
Ovulation rate	Lower	[11,34,38,39,40,41,42]
Ovum/ova quality	Lower	[31,38,43]
Conception rate	Lower due to the combined effects of the above factors	[16,19,22,44,45,46]
Early pregnancy loss	Higher due to lower embryo quality and impaired uterine environment	[11,39,40,42,47]
Pregnancy rate (ewes pregnant/ewes bred)	Lower due to the combined effects of the above factors	[16,45,46,48,49]
Number of lambs born per ewe bred	Lower due to the combined effects of the above factors	[11,16,19,41,45,46,50,51]
Gestation length	Shorter	[52,53]
Lamb birth weight	Lower	[19,20,45,51,54,55]
Colostrum production	Lower and composition differs	[51,56,57,58,59,60]
Milk production	Lower	[61,62,63,64]
Milk composition	Lower fat and protein percentage	[62,63]
Mothering ability	Some studies suggest poorer but not all	[50,65,66]
Lamb vigor at birth	Lower	[20]
Lamb survival	Lower due to above factors from birth	[16,20,42,45,65]
Lamb growth to weaning and weaning weight	Lower due to above factors from birth	[20,42,45,54,65]
Lambs weaned per ewe presented for breeding	Lower due to the combined effects of the above factors	[14,46,48]

## Data Availability

Not applicable.

## References

[1] Farrell L.J., Kenyon P.R., Morris S.T., Tozer P.R. (2020). The Impact of Hogget and Mature Flock Reproductive Success on Sheep Farm Productivity. Agriculture.

[2] Farrell L.J., Kenyon P.R., Tozer P.R., Morris S.T. (2021). Determining the Impact of Hogget Breeding Performance on Profitability under a Fixed Feed Supply Scenario in New Zealand. Animals.

[3] Young J.M., Thompson A.N., Kennedy A.J. (2010). Bioeconomic modelling to identify the relative importance of a range of critical control points for prime lamb production systems in south-west Victoria. Anim. Prod. Sci..

[4] Young J.M., Trompf J., Thompson A.N. (2014). The critical control points for increasing reproductive performance can be used to inform research priorities. Anim. Prod. Sci..

[5] Tocker J., Behrendt R., Raeside M., Malcolm B. (2020). The impact of ewe lamb mating and different feeding strategies over summer–autumn on profit and risk: A case study in south-west Victoria. Anim. Prod. Sci..

[6] Court J., Hides S., Webb-Ware J. (2010). Sheep Farming for Meat and Wool.

[7] Rattray P.V., Brookes I.M., Nicol A.M., Rattray P.V., Brookes I.M., Nicol A.M. (2017). Pastures and Supplements for Grazing Animals.

[8] Ferguson D.M., Schreurs N.M., Kenyon P.R., Jacob R.H. (2014). Balancing consumer and societal requirements for sheep meat production: An Australasian perspective. Meat Sci..

[9] Freer M., Dove H. (2002). Sheep Nutrition.

[10] Kenyon P.R., Thompson A.N., Morris S.T. (2014). Breeding ewe lambs successfully to improve lifetime performance. Small Rumin. Res..

[11] Edwards S.J., Juengel J.L. (2017). Limits on hogget lambing: The fertility of the young ewe. N. Z. J. Agric. Res..

[12] Sloane R. (2018). 2017 Merino Husbandry Practices Survey Final Report.

[13] Statistics New Zealand Infoshare. https://www.stats.govt.nz/tools/stats-infoshare.

[14] Dyrmundsson O.R. (1973). Puberty and early reproductive performance in sheep. I. Ewe lambs. Anim. Breed. Abstr..

[15] Rosales Nieto C.A., Thompson A.N., Martin G.B. (2018). A new perspective on managing the onset of puberty and early reproductive performance in ewe lambs: A review. Anim. Prod. Sci..

[16] Hutchison D., Clarke B.E., Hancock S., Thompson A.N., Bowen E., Jacobson C. (2022). Lower Reproductive Rate and Lamb Survival Contribute to Lower Lamb Marking Rate in Maiden Ewes Compared to Multiparous Ewes. Animals.

[17] Corner-Thomas R.A., Ridler A.L., Morris S.T., Kenyon P.R. (2015). Ewe lamb live weight and body condition scores affect reproductive rates in commercial flocks. N. Z. J. Agric. Res..

[18] Kenyon P.R., Pinchbeck G.L., Perkins N.R., Morris S.T., West D.M. (2004). Identifying factors which maximise the lambing performance of hoggets: A cross sectional study. N. Z. Vet. J..

[19] Corner R.A., Blair H.T., Morris S.T., Kenyon P.R. (2013). A comparison of aspects of the reproductive success of ewe lambs and mixed age ewes joined over the same period. Proc. N. Z. Soc. Anim. Prod..

[20] Pettigrew E.J., Hickson R.E., Blair H.T., Griffiths K.J., Ridler A.L., Morris S.T., Kenyon P.R. (2021). Differences in lamb production between ewe lambs and mature ewes. N. Z. J. Agric. Res..

[21] Hafez E.S. (1952). Studies on the breeding season and reproduction of the ewe Part I. The breeding season in different environments Part II. The breeding season in one locality. J. Agric. Sci..

[22] Smith J.F., Knight T.W., Fielden E.D., Smith J.F. (1998). Reproductive management of sheep. Reproductive Management of Grazing Ruminants in New Zealand.

[23] McMillan W., Parker W. (1981). Mating of Ewe Hoggets—An Appraisal for a Wairarapa Farm.

[24] Williams S.M. (1954). Fertility in Clun Forest sheep. J. Agric. Sci..

[25] Edey T.N., Kilgour R., Bremner K. (1978). Sexual-behavior and reproductive-performance of ewe lambs at and after puberty. J. Agric. Sci..

[26] Hare L., Bryant M.J. (1985). Ovulation rate and embryo survival in young ewes mated either at puberty or at the 2nd or 3rd estrus. Anim. Reprod. Sci..

[27] Schick G. (2001). Hogget mating—Will you follow the trend?. Wool Grow. Summer.

[28] Foote W., Sefidbakht N., Madsen M. (1970). Puberal estrus and ovulation and subsequent estrous cycle patterns in the ewe. J. Anim. Sci..

[29] Quirke J. (1978). Reproductive performance of Galway, Finnish Landrace and Finn-cross ewe lambs. Ir. J. Agric. Res..

[30] Edey T.N., Chu T.T., Kilgour R., Smith J.F., Tervit H.R. (1977). Estrus without ovulation in pubertal ewes. Theriogenology.

[31] Quirke J.F., Hanrahan J.P., Gosling J.P. (1981). Duration of estrus, ovulation rate, time of ovulation and plasma-lh, total estrogen and progesterone in galway adult ewes and ewe lambs. J. Reprod. Fertil..

[32] Chu T., Edey T. (1978). Reproductive Performance of Ewe Lambs at Puberty.

[33] Dyrmundsson O.R. (1981). Natural factors affecting puberty and reproductive-performance in ewe lambs—A review. Livest. Prod. Sci..

[34] Davies M.C.G., Beck N.F.G. (1993). A comparison of plasma prolactin, LH and progesterone concentrations during estrus and early-pregnancy in ewe lambs and ewes. Anim. Prod..

[35] Allison A.J., Kelly R.W., Lewis J.S., Binnie D.B. (1975). Preliminary studies on the efficiency of mating on ewe hoggets. Proc. N. Z. Soc. Anim. Prod..

[36] Kenyon P.R., Morris S.T., West D.M. (2010). Proportion of rams and the condition of ewe lambs at joining influences their breeding performance. Anim. Prod. Sci..

[37] Stevens D., McIntyre S. (1999). Hogget Mating Survey.

[38] Quirke J.F., Hanrahan J.P. (1977). Comparison of survival in uteri of adult ewes of cleaved ova from adult ewes and ewe lambs. J. Reprod. Fertil..

[39] Beck N.F.G., Davies M.C.G., Davies B. (1996). A comparison of ovulation rate and late embryonic mortality in ewe lambs and ewes and the role of late embryo loss in ewe lamb subfertility. Anim. Sci..

[40] Paganoni B.L., Ferguson M.B., Ferrio S., Jones C., Kearney G.A., Kenyon P.R., Macleay C., Vinoles C., Thompson A.N. (2014). Early reproductive losses are a major factor contributing to the poor reproductive performance of Merino ewe lambs mated at 8–10 months of age. Anim. Prod. Sci..

[41] Forrest P.A., Bichard M. (1974). Analysis of production records from a lowland sheep flock. 2. Flock statistics and reproductive-performance. Anim. Prod..

[42] Shorten P.R., Edwards S.J., Juengel J.L. (2021). The role of reproductive loss on flock performance: A comparison of nine industry flocks. Transl. Anim. Sci..

[43] McMillan W., McDonald M. (1985). Survival of fertilized ova from ewe lambs and adult ewes in the uteri of ewe lambs. Anim. Reprod. Sci..

[44] Keane M.G. (1976). Breeding from ewe lambs. Farm Food Res..

[45] Annett R.W., Carson A.F. (2006). Effects of plane of nutrition during the first month of pregnancy on conception rate, foetal development and lamb output of mature and adolescent ewes. Anim. Sci..

[46] Mulvaney F.J. (2011). Investigating methods to improve the reproductive performance of hoggets: A thesis presented in partial fulfilment of the requirements for the degree of Doctor of Philosophy in Animal Science at Massey University, Palmerston North, New Zealand. Ph.D. Thesis.

[47] O’Connell A.R., Demmers K.J., Smaill B., Reader K.L., Juengel J.L. (2016). Early embryo loss, morphology, and effect of previous immunization against androstenedione in the ewe. Theriogenology.

[48] Donald H.P., Read J.L., Russell W.S. (1968). A comparative trial of crossbred ewes by Finnish Landrace and other sires. Anim. Prod..

[49] Khan T.H., Beck N.F.G., Khalid M. (2007). The effects of GnRH analogue (buserelin) or hCG (Chorulon) on day 12 of pregnancy on ovarian function, plasma hormone concentrations, conceptus growth and placentation in ewes and ewe lambs. Anim. Reprod. Sci..

[50] Munoz C., Carson A.F., McCoy M.A., Dawson L.E.R., O’Connell N.E., Gordon A.W. (2009). Effect of plane of nutrition of 1-and 2-year-old ewes in early and mid-pregnancy on ewe reproduction and offspring performance up to weaning. Animal.

[51] Pattinson S., Davies D.A.R., Winter A. (1992). Colostrum and lamb production of prolific ewes. BSAP Occas. Publ..

[52] Baker R.L., Clarke J.N., Diprose G.D. (1981). Effect of mating Romney ewe hoggets on lifetime production—Preliminary results. N. Z. Soc. Anim. Prod..

[53] Quirke J.F., Hanrahan J.P. (1983). Comparison of the survival of fertilized-eggs from adult ewes in the uteri of adult ewes and ewe lambs. J. Reprod. Fertil..

[54] Gbangboche A., Adamou-Ndiaye M., Youssao A., Farnir F., Detilleux J., Abiola F., Leroy P. (2006). Non-genetic factors affecting the reproduction performance, lamb growth and productivity indices of Djallonke sheep. Small Rumin. Res..

[55] Gootwine E., Rozov A. (2006). Seasonal effects of birth weight of lambs born to prolific ewes maintained under intensive management. Livest. Sci..

[56] Campion F.P., Crosby T.F., Creighton P., Fahey A.G., Boland T.M. (2019). An investigation into the factors associated with ewe colostrum production. Small Rumin. Res..

[57] Halliday R. (1976). Variations in immunoglobulin concentrations in Finnish × Dorset Horn lambs. Res. Vet. Sci..

[58] Abd-Allah M. (2013). Effects of parity and nutrition plane during late pregnancy on metabolic responses, colostrum production and lamb output of Rahmani ewes. Egypt. J. Anim. Prod..

[59] Gilbert R., Gaskins C., Hillers J., Parker C., McGuire T. (1988). Genetic and environmental factors affecting immunoglobulin G1 concentrations in ewe colostrum and lamb serum. J. Anim. Sci..

[60] Argüello A., Castro N., Alvarez S., Capote J. (2006). Effects of the number of lactations and litter size on chemical composition and physical characteristics of goat colostrum. Small Rumin. Res..

[61] Morgan J.E., Fogarty N.M., Nielsen S., Gilmour A.R. (2006). Milk yield and milk composition from grazing primiparous non-dairy crossbred ewes. Aust. J. Agric. Res..

[62] Afolayan R., Fogarty N., Morgan J., Gaunt G., Cummins L., Gilmour A.R., Nielsen S. (2009). Genetic analysis of milk production and composition in crossbred ewes from different maternal genotypes. Anim. Prod. Sci..

[63] Peeters R., Buys N., Robijns L., Vanmontfort D., Van Isterdael J. (1992). Milk yield and milk composition of Flemish Milksheep, Suffolk and Texel ewes and their crossbreds. Small Rumin. Res..

[64] Snowder G.D., Knight A.D., Van Vleck L.D., Bromley C.M., Kellom T.R. (2001). Usefulness of subjective ovine milk scores: I. Associations with range ewe characteristics and lamb production. J. Anim. Sci..

[65] Corner R.A., Mulvaney F.J., Morris S.T., West D.M., Morel P.C.H., Kenyon P.R. (2013). A comparison of the reproductive performance of ewe lambs and mature ewes. Small Rumin. Res..

[66] Murphy P.M., Purvis I.W., Lindsay D.R., Le Neindre P., Orgeur P., Poindron P. (1994). Measures of temperament are highly repeatable in merino sheep and some are related to maternal behaviour. Proc. Aust. Soc. Anim. Prod..

[67] Fogarty N.M., Ingham V.M., Gilmour A.R., Afolayan R.A., Cummins L.J., Edwards J.E.H., Gaunt G.M. (2007). Genetic evaluation of crossbred lamb production. 5. Age of puberty and lambing performance of yearling crossbred ewes. Aust. J. Agric. Res..

[68] Ch’ang T., Rae A. (1970). The genetic basis of growth, reproduction, and maternal environment in Romney ewes. Aust. J. Agric. Res..

[69] Ch’Ang T., Rae A. (1972). The genetic basis of growth, reproduction, and maternal environment in Romney ewes. II.* Genetic covariation between hogget characters, fertility, and maternal environment of the ewe. Aust. J. Agric. Res..

[70] Alkass J.E., Aziz D.A., Al-Nidawi K.A. (1994). Genetic aspects of puberty in Awassi ewes. Small Rumin. Res..

[71] Baker R., Morris C. Selection for early puberty and increased fertility at first mating. Proceedings of the 2nd World Congress on Genetics Applied to Livestock Production.

[72] Meyer H.H., Bigham M.L., Baker R.L., Harvey T.G., Hickey S.M. (1994). Effects of Booroola Merino breeding and the FecB gene on performance of crosses with longwool breeds. 1. Effects on growth, onset of puberty, wool production and wool traits. Livest. Prod. Sci..

[73] Fossceco S.L., Notter D.R. (1995). Heritabilities and genetic correlations of body weight, testis growth and ewe lamb reproductive traits in crossbred sheep. Anim. Sci..

[74] Fogarty N., Brash L., Gilmour A. (1994). Genetic parameters for reproduction and lamb production and their components and liveweight, fat depth and wool production in Hyfer sheep. Aust. J. Agric. Res..

[75] Newton J.E., Brown D.J., Dominik S., van der Werf J.H.J. (2014). Genetic and phenotypic parameters between yearling, hoggetand adult reproductive performance and age of first oestrus in sheep. Anim. Prod. Sci..

[76] Rosales Nieto C.A., Ferguson M.B., Macleay C.A., Briegel J.R., Martin G.B., Thompson A.N. (2013). Selection for superior growth advances the onset of puberty and increases reproductive performance in ewe lambs. Anim. Int. J. Anim. Biosci..

[77] Rosales Nieto C.A., Ferguson M.B., Macleay C.A., Briegel J.R., Wood D.A., Martin G.B., Thompson A.N. (2013). Ewe lambs with higher breeding values for growth achieve higher reproductive performance when mated at age 8 months. Theriogenology.

[78] Thompson A., Bairstow C., Ferguson M., Kearney G., Macleay C., Thompson H., Paganoni B. (2019). Growth pattern to the end of the mating period influences the reproductive performance of merino ewe lambs mated at 7 to 8 months of age. Small Rumin. Res..

[79] Rosales Nieto C.A., Ferguson M.B., Macleay C.A., Briegel J.R., Wood D.A., Martin G.B., Bencini R., Thompson A.N. (2018). Milk production and composition, and progeny performance in young ewes with high merit for rapid growth and muscle and fat accumulation. Animal.

[80] Newton J.E., Brown D.J., Dominik S., van der Werf J.H.J. (2017). Impact of young ewe fertility rate on risk and genetic gain in sheep-breeding programs using genomic selection. Anim. Prod. Sci..

[81] Moore R.W., McMillan W.H., Dockrill G., Dow B.W. (1989). Lambing of Romney and Booroola cross hoggets with and without the F gene under different pasture allowances. N. Z. Soc. Anim. Prod..

[82] Kenyon P., Blair H. (2014). Foetal programming in sheep–effects on production. Small Rumin. Res..

[83] Walsh S.W., Mossa F., Butler S.T., Berry D.P., Scheetz D., Jimenez-Krassel F., Tempelman R.J., Carter F., Lonergan P., Evans A.C.O. (2014). Heritability and impact of environmental effects during pregnancy on antral follicle count in cattle. J. Dairy Sci..

[84] Bollwein H., Kawashima C., Shimizu T., Miyamoto A., Kaske M., Juengel J.L., Miyamoto A., Price C., Reynolds L.P., Smith M.F., Webb R. (2019). Impact of metabolism and production diseases on reproductive function in dairy cows. Reproduction in Domestic Ruminants VIII: Proceedings of the Ninth International Symposium on Reproduction in Domestic Ruminants.

[85] Dýrmundsson Ø.R. (1978). Studies on the breeding season of Icelandic ewes and ewe lambs. J. Agric. Sci..

[86] Fitzgerald J., Butler W.R. (1982). Seasonal Effects and Hormonal Patterns Related to Puberty in Ewe Lambs. Biol. Reprod..

[87] Foster D., Yellon S., Olster D.H. (1985). Internal and external determinants of the timing of puberty in the female. Reproduction.

[88] Rosales Nieto C.A., Ferguson M.B., Briegel J.R., Hedger M.P., Martin G.B., Thompson A.N. (2019). Pre-pubertal growth, muscle and fat accumulation in male and female sheep—Relationships with metabolic hormone concentrations, timing of puberty and reproductive outcomes. Reprod. Domest. Anim..

[89] Edwards S.J., Juengel J.L., O’Connell A.R., Johnstone P.D., Farquhar P.A., Davis G.H. (2015). Attainment of puberty by ewes in the first year of life is associated with improved reproductive performance at 2 years of age. Small Rumin. Res..

[90] Wall A., Juengel J., Edwards S., Rendel J. (2018). The economic value of replacement breeding ewes attaining puberty within their first year of life on New Zealand sheep farms. Agric. Syst..

[91] Quirke J.F. (1981). Regulation of puberty and reproduction in female lambs: A review. Livest. Prod. Sci..

[92] Thompson A.N., Bowen E., Keiller J., Pegler D., Kearney G., Rosales-Nieto C.A. (2021). The Number of Offspring Weaned from Ewe Lambs Is Affected Differently by Liveweight and Age at Breeding. Animals.

[93] Bunge R., Thomas D.L., Nash T.G. (1993). Performance of hair breeds and prolific wool breeds of sheep in southern Illinois: Lamb production of F1 ewe lambs. J. Anim. Sci..

[94] Knights M., Baptiste Q.S., Lewis P.E. (2002). Ability of ram introduction to induce LH secretion, estrus and ovulation in fall-born ewe lambs during anestrus. Anim. Reprod. Sci..

[95] Kenyon P.R., Vinoles C., Morris S.T. (2012). Effect of teasing by the ram on the onset of puberty in Romney ewe lambs. N. Z. J. Agric. Res..

[96] Dýrmundsson Ó.R., Lees J.L. (1972). Effect of rams on the onset of breeding activity in Clun Forest ewe lambs. J. Agric. Sci..

[97] Cave L.M., Kenyon P.R., Morris S.T. (2012). Effect of timing of exposure to vasectomised rams and ewe lamb body condition score on the breeding performance of ewe lambs. Anim. Prod. Sci..

[98] Kenyon P.R., Morel P.C.H., Morris S.T., Burnham D.L., West D.M. (2007). Effect of the ratio of teaser rams used prior to breeding on the reproductive performance of ewe hoggets. N. Z. Vet. J..

[99] Kenyon P.R., Morel P.C.H., Morris S.T., Burnham D.L., West D.M. (2006). The effect of length of use of teaser rams prior to mating and individual liveweight on the reproductive performance of ewe hoggets. N. Z. Vet. J..

[100] Kenyon P.R., Morris S.T., West D.M. (2008). Can Romney ram lambs whose scrotums had been shortened by the use of a rubber ring be used as an alternative to vasectomised Perendale rams for inducing early breeding activity in Romney ewe lambs?. N. Z. Vet. J..

[101] Corner-Thomas R.A., Hickson R.E., Morris S.T., Kenyon P.R. (2014). The influences of live weight and body condition score of ewe lambs from breeding to lambing on the live weight of their singleton lambs to weaning. Small Rumin. Res..

[102] Haslin E., Corner-Thomas R.A., Kenyon P.R., Pettigrew E.J., Hickson R.E., Morris S.T., Blair H.T. (2022). Effect of Breeding Heavier Romney Ewe Lambs at Seven Months of Age on Lamb Production and Efficiency over Their First Three Breeding Seasons. Animals.

[103] Kenyon P.R., Maloney S.K., Blache D. (2014). Review of sheep body condition in relation to production characteristics. N. Z. J. Agric. Res..

[104] Owens F.N., Dubeski P., Hanson C.F. (1993). Factors that alter the growth and development of ruminants. J. Anim. Sci..

[105] Rosales Nieto C., Ferguson M., Thompson H., Briegel J., Macleay C., Martin G., Thompson A. (2015). Relationships among Puberty, Muscle and Fat, and Liveweight Gain during Mating in Young Female Sheep. Reprod. Domest. Anim..

[106] Kenyon P.R. (2012). Hogget Performance: Unlocking the Potential.

[107] Kenyon P.R., Corner-Thomas R.A., Paganoni B.L., Morris S.T. (2014). Percentage of Mature Liveweight Affects Reproductive Performance in Ewe Lambs. Proc. Aust. Soc. Anim. Prod..

[108] Evans A.C.O. (2003). Ovarian follicle growth and consequences for fertility in sheep. Anim. Reprod. Sci..

[109] Bichard M., Younis A.A., Forrest P.A., Cumberla P. (1974). Analysis of production records from a lowland sheep flock. 4. Factors influencing incidence of successful pregnancy in young females. Anim. Prod..

[110] Edwards S.J., Smaill B., O’Connell A.R., Johnstone P.D., Stevens D.R., Quirke L.D., Farquhar P.A., Juengel J.L. (2016). Reduced ovulation rate, failure to be mated and fertilization failure/embryo loss are the underlying causes of poor reproductive performance in juvenile ewes. Anim. Reprod. Sci..

[111] Corner-Thomas R.A., Panns J.M., Kemp P.D., Morris S.T., Kenyon P.R. (2017). The effect of grazing ewe lambs on lucerne (Medicago sativa) prior to breeding on aspects of reproductive performance. Proc. N. Z. Soc. Anim. Prod..

[112] Mulvaney F.J., Morris S.T., Kenyon P.R., West D.M., Morel P.C.H. (2010). Effect of liveweight at the start of the breeding period and liveweight gain during the breeding period and pregnancy on reproductive performance of hoggets and the liveweight of their lambs. N. Z. J. Agric. Res..

[113] Dyrmundsson O.R., Lees J.L. (1972). Effect of autumn shearing on breeding activity in Clun Forest ewe lambs. J. Agric. Sci..

[114] Dwyer C., Ferguson D.M., Lee C., Fisher A. (2017). Reproductive management (including impacts of prenatal stress on offspring development). Advances in Sheep Welfare.

[115] Kenyon P.R., Morel P.C.H., Morris S.T., West D.M. (2007). Effect of the age of rams on reproductive performance of ewe hoggets. N. Z. Vet. J..

[116] Kenyon P.R., Smith S.L., Morel P.C.H., Morris S.T., West D.M. (2009). The effect of the maturity and prior breeding activity of rams and body condition score of ewe hoggets on the reproductive performance of ewe hoggets. N. Z. Vet. J..

[117] Wallace J.M., Aitken R.P., Cheyne M.A. (1996). Nutrient partitioning and fetal growth in rapidly growing adolescent ewes. J. Reprod. Fertil..

[118] Wallace J.M. (2000). Nutrient partitioning during pregnancy: Adverse gestational outcome in overnourished adolescent dams. Proc. Nutr. Soc..

[119] Wallace J.M., Milne J.S., Aitken R.P. (2010). Effect of Weight and Adiposity at Conception and Wide Variations in Gestational Dietary Intake on Pregnancy Outcome and Early Postnatal Performance in Young Adolescent Sheep1. Biol. Reprod..

[120] Wallace J.M., Aitken R.P., Milne J.S., Hay W.W. (2004). Nutritionally mediated placental growth restriction in the growing adolescent: Consequences for the fetus. Biol. Reprod..

[121] Wallace J.M., Bourke D.A., Aitken R.P., Milne J.S., Hay W.W. (2003). Placental glucose transport in growth-restricted pregnancies induced by overnourishing adolescent sheep. J. Physiol. Lond..

[122] Wallace J.M. (2019). Competition for nutrients in pregnant adolescents: Consequences for maternal, conceptus and offspring endocrine systems. J. Endocrinol..

[123] Kenyon P.R., Morris S.T., Burnham D.L., West D.M. (2008). Effect of nutrition during pregnancy on hogget pregnancy outcome and birthweight and liveweight of lambs. N. Z. J. Agric. Res..

[124] Morris S.T., Kenyon P.R., West D.M. (2005). Effect of hogget nutrition in pregnancy on lamb birthweight and survival to weaning. N. Z. J. Agric. Res..

[125] Mulvaney F., Morris S., Kenyon P., West D., Morel P. (2010). Effect of nutrition around the time of breeding and during pregnancy on yearling liveweight change, pregnancy loss and live weight and survival of their offspring. Proc. N. Z. Soc. Anim. Prod..

[126] Clune T., Lockwood A., Hancock S., Thompson A.N., Beetson S., Campbell A.J.D., Glanville E., Brookes D., Trengove C., O’Handley R. (2022). Abortion and Lamb Mortality between Pregnancy Scanning and Lamb Marking for Maiden Ewes in Southern Australia. Animals.

[127] Mulvaney F.J., Kenyon P.R., Morris S.T., West D.M. (2008). Ewe lamb nutrition during pregnancy affects pregnancy outcome. Aust. J. Exp. Agric..

[128] Swanson T.J., Hammer C.J., Luther J.S., Carlson D.B., Taylor J.B., Redmer D.A., Neville T.L., Reed J.J., Reynolds L.P., Caton J.S. (2008). Effects of gestational plane of nutrition and selenium supplementation on mammary development and colostrum quality in pregnant ewe lambs. J. Anim. Sci..

[129] Ridler A.L., Vallee E., Corner R.A., Kenyon P.R., Heuer C. (2015). Factors associated with fetal losses in ewe lambs on a New Zealand sheep farm. N. Z. Vet. J..

[130] Ridler A., Corner-Thomas R., Kenyon P., Griffiths K. (2017). Investigation of fetal loss in ewe lambs in relation to liveweight changes and progesterone concentrations in early to mid gestation. N. Z. Vet. J..

[131] Griffiths K.J., Ridler A.L., Heuer C., Corner-Thomas R.A., Kenyon P.R. (2016). The effect of liveweight and body condition score on the ability of ewe lambs to successfully rear their offspring. Small Rumin. Res..

[132] Mulvaney F.J., Morris S.T., Kenyon P.R., Morel P.C.H., West D.M. (2012). Effect of nutrition from mid-pregnancy to parturition on the live weight of twin-bearing hoggets and the live weight and survival of their lambs. N. Z. J. Agric. Res..

[133] Schreurs N.M., Kenyon P.R., Mulvaney F.J., Morel P.C.H., West D.M., Morris S.T. (2010). Response of additional ewe lamb liveweight during gestation on birth and weaning weight of offspring and liveweight of the ewe lamb at weaning. Anim. Prod. Sci..

[134] West D.M., Pomroy W.E., Collett M.G., Hill F.I., Ridler A.L., Kenyon P.R., Morris S.T., Pattison R.S. (2006). A possible role for Neospora caninum in ovine abortion in New Zealand. Small Rumin. Res..

[135] Howe L., West D.M., Collett M.G., Tattersfield G., Pattison R.S., Pomroy W.E., Kenyon P.R., Morris S.T., Williamson N.B. (2008). The role of Neospora caninum in three cases of unexplained ewe abortions in the southern North Island of New Zealand. Small Rumin. Res..

[136] Mulvaney F.J., Morris S.T., Kenyon P.R., Morel P.C.H., West D.M. (2010). Effect of nutrition pre-breeding and during pregnancy on breeding performance of ewe lambs. Anim. Prod. Sci..

[137] Clune T., Lockwood A., Hancock S., Thompson A., Beetson S., Campbell A. On-farm investigation of foetal and lamb losses in maiden ewes. Proceedings of the Sheep, Camelid and Goat Veterinarians Conference (Australian Veternary Association).

[138] Ridler A. Fetal Loss in Maiden Ewes—An Update.

[139] West D. (2002). Ovine abortion in New Zealand. N. Z. Vet. J..

[140] Kenyon P.R., Morris S.T., Revell D.K., McCutcheon S.N. (2003). Shearing during pregnancy—Review of a policy to increase birthweight and survival of lambs in New Zealand pastoral farming systems. N. Z. Vet. J..

[141] Kenyon P.R., Sherlock R.G., Morris S.T., Morel P.C.H. (2006). The effect of mid- and late-pregnancy shearing of hoggets on lamb birthweight, weaning weight, survival rate, and wool follicle and fibre characteristics. Aust. J. Agric. Res..

[142] Mulvaney F.J., Morris S.T., Kenyon P.R., Morel P.C.H., West D.M., Vinoles C., Glover K.M.M. (2013). Comparison between the reproductive performance of ewe hoggets and mature ewes following a progesterone based oestrus synchronization protocol. N. Z. J. Agric. Res..

[143] Ridler A.L., Flay K.J., Kenyon P.R., Blair H.T., Corner-Thomas R.A., Pettigrew E.J. (2022). Factors Associated with Mortality of Lambs Born to Ewe Hoggets. Animals.

[144] Hinch G.N., Brien F. (2014). Lamb survival in Australian flocks: A review. Anim. Prod. Sci..

[145] McMillan W.H. (1983). Hogget lamb mortality. Proc. N. Z. Soc. Anim. Prod..

[146] Stevens D.R. (2010). On-farm ewe lamb mating outcomes from feeding practices before mating and during pregnancy. Proc. N. Z. Soc. Anim. Prod..

[147] Jacobson C., Bruce M., Kenyon P.R., Lockwood A., Miller D., Refshauge G., Masters D.G. (2020). A review of dystocia in sheep. Small Rumin. Res..

[148] Dwyer C.M., Conington J., Corbiere F., Holmøy I.H., Muri K., Nowak R., Rooke J., Vipond J., Gautier J.M. (2016). Invited review: Improving neonatal survival in small ruminants: Science into practice. Animal.

[149] Nowak R., Poindron P. (2006). From birth to colostrum: Early steps leading to lamb survival. Reprod. Nutr. Dev..

[150] Corner-Thomas R.A., Cranston L.M., Kemp P.D., Morris S.T., Kenyon P.R. (2018). The influence of three herbage types on the liveweight change of twin-bearing hoggets and their lambs. N. Z. J. Agric. Res..

[151] Corner-Thomas R.A., Kemp P.D., Morris S.T., Kenyon P.R. (2014). Grazing alternative herbages in lactation increases the liveweight of both ewe lambs and their progeny at weaning. Anim. Prod. Sci..

[152] Mulvaney F., Morris S., Kenyon P., West D., Morel P. (2009). The effect of weaning at 10 or 14 weeks of age on liveweight changes in the hogget and her lambs. Proc. N. Z. Soc. Anim. Prod..

[153] Thomson B.C., Smith N.B., Muir P.D. (2021). Effect of birth rank and age at first lambing on lifetime performance and ewe efficiency. N. Z. J. Agric. Res..

[154] Tyrrell R.N. (1976). Some effects of pregnancy in 8-month-old Merino ewes. Aust. J. Exp. Agric..

[155] McCall D.G., Hight G.K. (1981). Environmental influences on hogget lambing performance and the relationship between hogget and two-tooth lambing performance. N. Z. J. Agric. Res..

[156] Flay K.J., Ridler A.L., Compton C.W.R., Kenyon P.R. (2021). Ewe Wastage in New Zealand Commercial Flocks: Extent, Timing, Association with Hogget Reproductive Outcomes and BCS. Animals.

[157] Kenyon P.R., van der Linden D.S., West D.M., Morris S.T. (2011). The effect of breeding hoggets on lifetime performance. N. Z. J. Agric. Res..

[158] Haslin E., Corner-Thomas R.A., Kenyon P.R., Pettigrew E.J., Hickson R.E., Morris S.T., Blair H.T. (2022). Effects of heavier live weight of ewe lambs at mating on fertility, lambing percentage, subsequent live weight and the performance of their progeny. N. Z. J. Agric. Res..

[159] Haslin E., Corner-Thomas R.A., Kenyon P.R., Pettigrew E.J., Hickson R.E., Morris S.T., Blair H.T. (2022). Breeding heavier ewe lambs at seven months of age did not impact their subsequent two and three-year-old ewe live weight and reproductive performance. N. Z. J. Agric. Res..

[160] Craig R.L. (1982). Breeding from Romney ewe hoggets in the Waihora group breeding scheme. N. Z. J. Agric. Sci..

[161] Loureiro M.F.P., Pain S.J., Kenyon P.R., Blair H.T. (2010). Do fetuses from primiparous one-year-old ewes differ from those of multiparous mature ewes?. Proc. N. Z. Soc. Anim. Prod..

[162] Loureiro M.F.P., Pain S.J., Kenyon P.R., Peterson S.W., Blair H.T. (2012). Single female offspring born to primiparous ewe-lambs are lighter than those born to adult multiparous ewes but their reproduction and milk production are unaffected. Anim. Prod. Sci..

[163] Pettigrew E., Hickson R., Morris S., Lopez-Villalobos N., Pain S., Kenyon P., Blair H. (2019). The effects of birth rank (single or twin) and dam age on the lifetime productive performance of female dual purpose sheep (Ovis aries) offspring in New Zealand. PLoS ONE.

[164] Pettigrew E., Hickson R., Morris S., Kenyon P., Corner-Thomas R., Haslin E., Blair H. (2021). The Effect of Age of Dam and Birth Rank on the Reproductive Performance of Ewes as One- and Two-Year-Olds. Animals.

